# The Effect of the Retroperitoneal Transpsoas Minimally Invasive Lateral Interbody Fusion on Segmental and Regional Lumbar Lordosis

**DOI:** 10.1100/2012/516706

**Published:** 2012-08-02

**Authors:** Tien V. Le, Andrew C. Vivas, Elias Dakwar, Ali A. Baaj, Juan S. Uribe

**Affiliations:** Department of Neurosurgery and Brain Repair, University of South Florida, 2 Tampa General Circle, 7th Floor, Tampa, FL 33606, USA

## Abstract

*Background*. The minimally invasive lateral interbody fusion (MIS LIF) in the lumbar spine can correct coronal Cobb angles, but the effect on sagittal plane correction is unclear. *Methods*. A retrospective review of thirty-five patients with lumbar degenerative disease who underwent MIS LIF without supplemental posterior instrumentation was undertaken to study the radiographic effect on the restoration of segmental and regional lumbar lordosis using the Cobb angles on pre- and postoperative radiographs. Mean disc height changes were also measured. *Results*. The mean follow-up period was 13.3 months. Fifty total levels were fused with a mean of 1.42 levels fused per patient. Mean segmental Cobb angle increased from 11.10° to 13.61° (*P* < 0.001) or 22.6%. L2-3 had the greatest proportional increase in segmental lordosis. Mean regional Cobb angle increased from 52.47° to 53.45° (*P* = 0.392). Mean disc height increased from 6.50 mm to 10.04 mm (*P* < 0.001) or 54.5%. *Conclusions*. The MIS LIF improves segmental lordosis and disc height in the lumbar spine but not regional lumbar lordosis. Anterior longitudinal ligament sectioning and/or the addition of a more lordotic implant may be necessary in cases where significant increases in regional lumbar lordosis are desired.

## 1. Introduction

Minimally invasive spine surgery is an alternative to traditional open operations for the treatment of degenerative spine disease. Advantages include less major complications, less blood loss, less wound infections, earlier patient mobilization, and shorter hospital stays [1–7]. 

Minimally invasive lateral interbody fusion (MIS LIF), such as, with Extreme Lateral Interbody Fusion (XLIF; NuVasive, San Diego, CA, USA) or Direct Lateral Interbody Fusion (DLIF; Medtronic, Minneapolis, MN, USA), has been used to treat degenerative spine disease, including degenerative scoliosis [4–8]. In the lumbar spine, a retroperitoneal transpsoas approach is taken. Using this technique, coronal Cobb angles can be improved [5–7, 9]. The effects of sagittal Cobb angles, such as, with lumbar lordosis (LL) and the overall global sagittal balance have not been as well established, however [9, 10]. This is an important topic since a positive global sagittal imbalance is most closely linked to a decreased quality of life, health status outcomes, and function [11]. Sagittal imbalance can lead to higher energy requirements to stand and ambulate, leading to early fatigue, intolerance to standing, and walking with compensation through other joints.

The aim of this study is to evaluate the effect of the XLIF technique in the lumbar spine on the restoration of segmental and regional LL in patients with degenerative spine disease. An additional study focus will be to evaluate the effect on segmental disc heights in the sagittal plane.

## 2. Materials and Methods

This is an IRB-approved, retrospective review of a prospectively collected database. Thirty-five consecutive patients with available preoperative and postoperative radiographs for analysis were included in this study (Table 1). The mean age at the time of surgery was 61.3 years. All patients had evidence of lumbar degenerative disease (spondylosis, adult degenerative scoliosis, or adjacent segment failure). 

To be included, patients had to have undergone MIS LIF with placement of a 10° lordotic, PEEK interbody cage at any level from L1-2–L4-5 without any supplemental posterior instrumentation. Specifically, only patients who underwent stand-alone interbody fusions, patients who had interbody fusions supplemented only with a lateral plate, or patients who underwent interbody fusion where only the caudal level was instrumented from a previous operation (pedicle screws, facet screws, or interspinous process spacers) were measured for sagittal segmental and regional Cobb angles as well as disc heights. 

Surgical indications included segmental instability for the target disc with combined minimal canal stenosis, degenerative disc disease, disc herniation, and adjacent segment failure.

The XLIF procedure was performed as previously described [8]. Patients are positioned in a lateral decubitus position, typically with the side giving the best clearance of the ipsilateral iliac crest or the concave side of any scoliotic curve up. A small incision is made, and a muscle splitting technique is used to gain access to the retroperitoneal space and facilitate localization of the correct disc space under fluoroscopic guidance. 

A discectomy is performed, endplates prepared, and a 10° lordotic, PEEK cage (CoRoent XL, NuVasive, San Diego, CA, USA) of either 50, 55, or 60 mm in length, 18 or 22 mm in width, and 8 to 10 mm in height was implanted. All cages were filled with allograft [0.7–1.4 mg of recombinant human bone morphogenetic protein-2 (rhBMP-2)(INFUSE, Medtronic, Minneapolis, MN, USA) mixed with hydroxyapatite and tricalcium phosphate (Formagraft, NuVasive, San Diego, CA, USA) per level] or 5 cc of cadaveric cancellous bone mixed with mesenchymal stem cells (Osteocel, NuVasive, San Diego, CA). Implants were centered just posterior to half of the disc space. The ALL and PLL were left intact.

**Figure 1:**
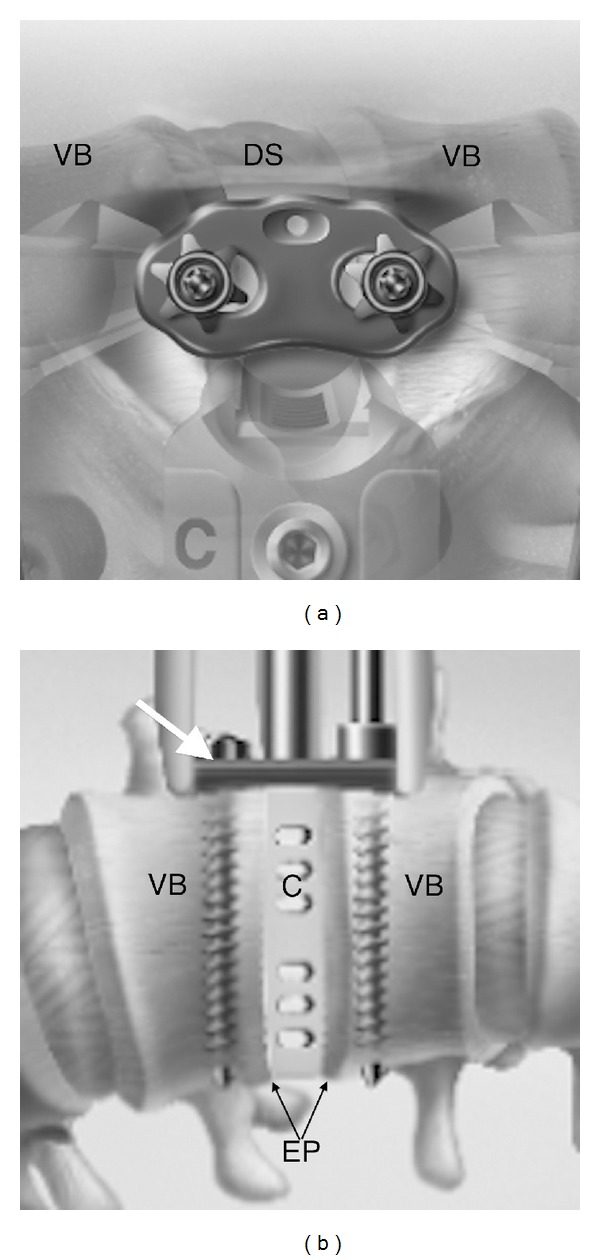
XLP lateral plate. (a) Lateral view. Notice the plate spans across the disc space (DS) and is secured down to the vertebral bodies (VB) with lock nuts. (b) AP view. Lateral plate (white arrow) is seated on two bicortical screws, which are parallel to the adjacent endplates (EP). A cage (C) is depicted in the disc space. (Images used with permission of NuVasive, Inc., San Diego, CA, USA).

A 2-screw fixation (one rostral and one caudal) titanium lateral plate (XLP, NuVasive, San Diego, CA, USA) was used in all but one patient (Figure 1). Appropriate positioning and size were fluoroscopically confirmed. The rostral and caudal screw entry points were centered to clear each corresponding endplate as well as the ipsilateral segmental artery. Screws were placed parallel to the endplates, and bicortical purchase was obtained. The plate was then seated over the screw heads, and the lock nuts were secured. 

Preoperative and postoperative upright anterior-posterior and lateral lumbar spine radiographs were obtained in all patients. The most recent postoperative radiographs from routine 6- and 12-week, 6-, 12-, 18-, and 24-month follow-up appointments were used for comparison.

**Figure 2:**
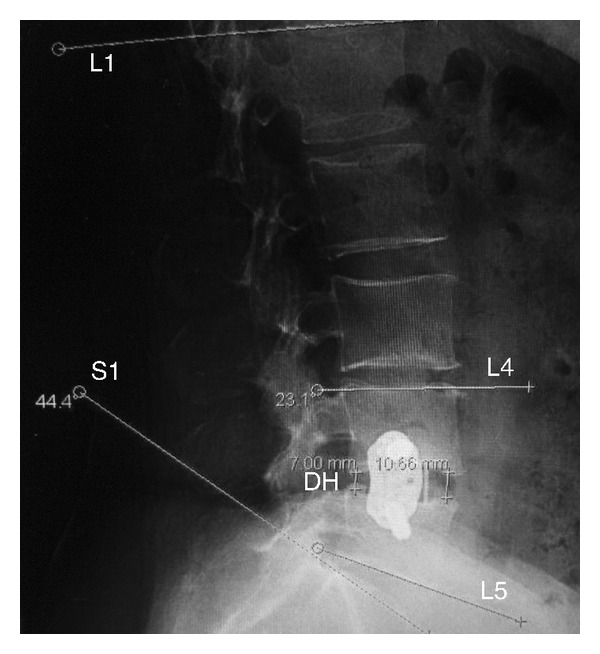
Representative lordosis and disc height measurements. Regional Cobb angles are based on the superior endplate of L1 and the superior endplate of S1 to measure regional lumbar lordosis. Segmental Cobb angles are based on the superior endplate of the rostral vertebral body and the inferior endplate of the caudal vertebral body (L4 and L5 in this example). Disc heights are calculated using the average between the anterior and posterior disc heights.

Lordosis measurements were made on lateral radiographs. The Cobb method was used for segmental and regional LL measurements (Figure 2), [12, 13] All measurements were obtained digitally using Centricity 3.0 workstations (GE Healthcare). Segmental Cobb angles were measured using the superior endplate of the rostral vertebral body and inferior endplate of the caudal vertebral body. By using this method, measurements of the true angle can be obtained as opposed to a measurement of what may represent the lordosis of the cage. The mean disc height was taken as the mean of the anterior and posterior disc heights. 

All measurements were collected and organized using an excel spreadsheet (Microsoft, Redmond, WA, USA). Of the total, a hypolordosis subgroup (preoperative regional Cobb angle of <42°) and a normolordosis group (preoperative regional Cobb angle of ≥42°) were then analyzed for the above endpoints. Statistical analysis was carried out with IBM SPSS 19.0 using the paired *t*-test and nonparametric Wilcoxon Signed Ranks test.

## 3. Results

Thirty-five patients were included, of which 7 were hypolordotic and 28 were normolordotic based on preoperative lateral radiographs. The mean follow-up period was 13.3 months. Fifty total levels were fused giving a mean of 1.42 levels fused per patient. 

**Figure 3:**
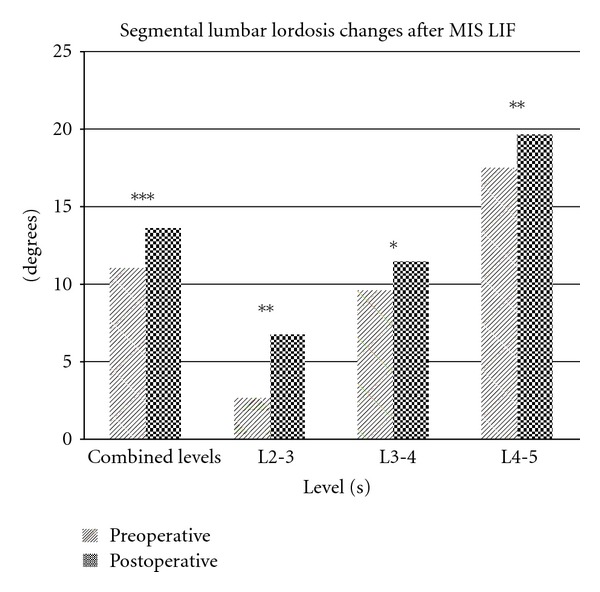
Segmental lumbar lordosis changes after MIS LIF. Statistically significant increases were observed at each measured level as well as in aggregate. (* = *P* < 0.05,** = *P* < 0.01,*** = *P* < 0.001).

**Figure 4:**
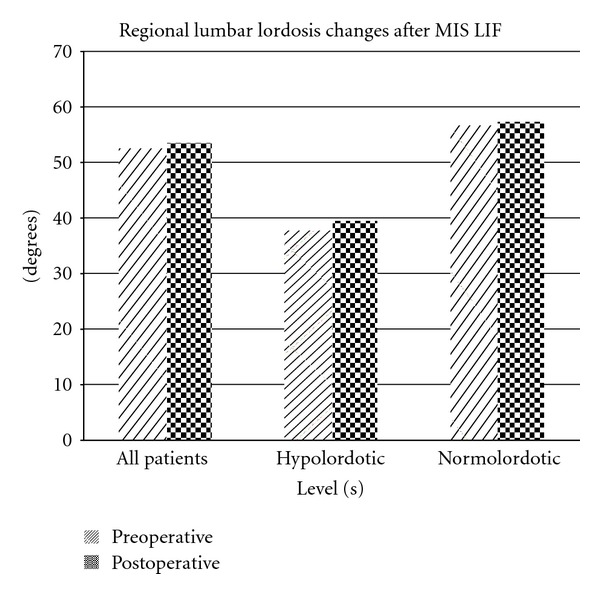
: Regional lumbar lordosis changes after MIS LIF. No statistically significant increases were observed.

**Figure 5:**
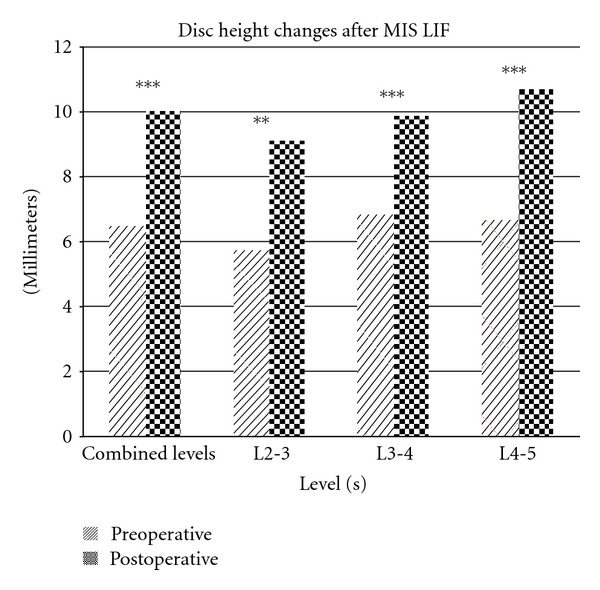
Disc height changes after MIS LIF. Statistically significant increases were observed at each measured level as well as in aggregate. (* = *P* < 0.05,** = *P* < 0.01,*** = *P* < 0.001).

 The mean segmental Cobb angle increased from 11.10° ± 9.29 to 13.61° ± 8.46 (*P* < 0.001) (Figure 3). The mean regional Cobb angle increased from 52.47° ± 10.55 to 53.45° ± 11.90 (*P* = 0.392) (Figure 4). The mean disc height increased from 6.50 mm ± 2.51 to 10.04 mm ± 2.75 (*P* < 0.001) (Figure 5). 

The proportional increase in mean segmental Cobb angle was 22.6% for all levels. Proportional gains in segmental Cobb angles progressively declined with more caudal lumbar segments, with 157.8%, 13.9%, and 8.7% increases for L2-3, L3-4, and L4-5, respectively.

The proportional increase in mean preoperative disc heights was 54.5% for all levels. A proportional increase in mean preoperative disc heights of 58.6%, 44.7%, and 61.0% was observed for L2-3, L3-4, and L4-5, respectively. 

For the hypolordotic subgroup, the mean segmental Cobb angle increased from 2.38° ± 8.61 to 5.90° ± 7.06 (*P* = 0.051). The mean regional Cobb angle increased from 37.74° ± 2.74 to 39.39° ± 10.53 (*P* = 0.636). The mean preoperative disc height increased from 6.45 mm ± 2.76 to 9.82 mm ± 3.25 (*P* < 0.043).

For the normolordotic subgroup, the mean segmental Cobb angle increased from 13.02° ± 8.37 to 15.30° ± 7.84 (*P* < 0.001). The mean regional Cobb angle increased from 56.40° ± 8.21 to 57.34° ± 9.52 (*P* = 0.498). The mean preoperative disc height increased from 6.51 mm ± 2.49 to 10.08 mm ± 2.68 (*P* < 0.001).

## 4. Discussion

The MIS LIF via the retroperitoneal transpsoas lumbar interbody fusion is an alternative to traditional open anterior-only, posterior-only, or circumferential operations [8]. Though the most common complications associated with this procedure include transient ipsilateral thigh numbness and iliopsoas weakness, in general, major complications are lower, there tends to be less blood loss, less wound infections, patients mobilize earlier, and hospital stays are shorter [1–7]. Clinical outcomes data are also promising as reported by Mundis et al. [10], where they demonstrated improved radiographic parameters as well as improved clinical results with a lower complication profile compared to traditional open approaches. 

Traditional open operations, such as, anterior lumbar interbody fusion (ALIF), posterior lumbar interbody fusion (PLIF), and transforaminal lumbar interbody fusion (TLIF) have led to the development of this technique. Briefly, advantages of the ALIF include a large interbody graft for disc space reexpansion, restoration of LL, and elimination of discogenic pain [14]. In addition, posterior facet joint complexes and tension bands remain intact. However, an access surgeon may be needed, and complications can include a risk of vascular injury and also rare iatrogenic retrograde ejaculation in males postoperatively. The TLIF[15, 16] was developed as a modification of the PLIF [17] to decrease the degree of nerve root and thecal sac manipulation, and it allows for interbody fusion, concurrent posterior segmental instrumentation, and circumferential fusion. Potential restoration of LL is gained by shortening of the posterior aspect of the spine by applying compressive forces to the segmental pedicle screws. It can be performed either in an open or minimally invasive manner. The graft size is typically smaller than that of the ALIF, however. 

Hsieh et al. [18] compared the postoperative radiographic changes of disc height, foraminal height, local (segmental) disc angle, and LL for ALIF and TLIF. Though both involve placement of an interbody graft and subsequently an increase in disc height, ALIF was found to be superior to TLIF in its capacity to restore foraminal height (18.5% increase versus 0.4% decrease), local disc angle (8.3° increase versus 0.1° decrease), and LL (6.2° increase versus 2.1° decrease). 

The MIS LIF via the retroperitoneal transpsoas approach shares the advantages of these two traditional approaches since large interbody cages can be placed to provide indirect decompression as in ALIF, and the operation can be done through a small incision in a minimally disruptive approach as in TLIF. 

This technique is also an appealing option for potential restoration of coronal and sagittal balance in spinal deformity due to the large, lordotic interbody cages, and the potential for less complications, given that traditional open posterior surgery for deformity has a 25% to 80% risk of postoperative complications including excessive blood loss, infection, neurologic injury, and medical complications [2, 3].

Of the radiographic spinopelvic parameters, a positive global sagittal imbalance, determined by the sagittal vertical axis (SVA) [19] or T1-SI [20], is most closely linked to decreased quality of life and health status outcomes. Specifically, patients with an SVA of >50 mm or a T1-SI of >0° can experience a significant decline in function [11]. These patients tend to have higher energy requirements to stand and ambulate, leading to early fatigue, intolerance to standing, and walking with compensation through other joints.

Regional LL is directly related to global sagittal alignment [10]. Multiple studies in asymptomatic adults have found the normal range of LL to be 42° to 66°.[21–26] There is clearly a wide range of what is considered normal. In addition to regional LL, segmental LL is not uniform, with the two most caudal motion segments accounting for up to 64% of LL [27–29]. Segmental lordosis progressively increases with more caudal segments, with 4°, 9°, 14°, 24°, and 24° of lordosis at L1-2, L2-3, L3-4, L4-5, and L5-S1, respectively. Overall, loss of lordosis is poorly tolerated in the lumbar spine [30, 31], and its maintenance or restoration is a critical surgical goal in order to better achieve global sagittal balance.

Acosta et al. [32] recently reported that segmental LL can be increased but not regional LL or global sagittal alignment in their series of 36 patients who underwent DLIF, of which 35 had supplemental posterior instrumentation. Their study group was heterogeneous including seven degenerative scoliosis patients. Some limitations of their study were that all but one patient had supplemental posterior instrumentation, 6° lordotic cages were used, segmental Cobb angle measurements were based on the endplates adjacent to the cage, and immediate postoperative radiographs were used for comparison.

Without similar limitations, the current study confirmed that MIS LIF can increase segmental lordosis and disc heights significantly but not regional lordosis. Only patients with degenerative lumbar spondylosis or evidence of adjacent segment failure who underwent lumbar MIS LIF using a 10° lordotic cage without any supplemental posterior instrumentation were included. This point is particularly important since prone positioning alone can potentially increase lordosis [33, 34]. The addition of pedicle screws and a precontoured rod, particularly if in a percutaneous fashion, can then confound the picture further, since, depending on the contouring, there can be a decrease or an increase of any gained lordosis with the cage placement alone [32, 34].

A lateral plate was acceptable since biomechanical studies have demonstrated its motion restriction in lateral bending and rotation but minimal influence on flexion and extension [35, 36]. This allows for a more clear investigation of only the implant's effect on segmental and regional LL, without any confounding effect of posterior instrumentation, especially when evaluating radiographs several months to years in followup. 

As opposed to the immediate postoperative radiograph, the most recent follow-up radiographs were used for comparison in this study. The use of the immediate postoperative radiograph would not allow enough time to see contributions of potential subsidence and/or collapse of the anterior support, leading to a potential overestimation of the correction gained in the long term [37]. 

Our segmental Cobb angles were measured using the superior endplate of rostral vertebral body and inferior endplate of caudal vertebral body as opposed to using endplates adjacent to the cage. By using this method, radiographic visualization is improved, especially with long-term followup and the addition of a lateral plate. More importantly, measurements of the true angle can be obtained as opposed to a measurement of what may actually represent the lordosis of the cage itself instead. 

The findings from this study underscore the potential role that MIS LIF has in spinal deformity surgery, given its advantages as discussed above. The large, lordotic interbody cages alone appear to account for increased segmental Cobb angle and disc height based on our results. Thus, it is reasonable to expect an even more robust LL restoration and improvement with even more lordotic cages if the tension of the anterior longitudinal ligament (ALL) was electively sectioned 

The greatest proportional increase in segmental LL was observed at L2-3, and this progressively decreased with lower lumbar segments. Given a constant cage lordosis and a progressively increasing physiologic segmental LL, this finding is not too surprising. Also, L1-2 and L2-3 are most amenable to correction in the coronal and sagittal planes [10], possibly because the normal lordosis is 4° and 9°, respectively. 

The regional lordosis did not significantly increase when looking at the study group as a whole or when comparing hypolordotic versus normolordotic subgroups. Potential explanations for this include the maintenance of the posterior facet complex and ALL, hypertrophied facet joints in degenerative disease, and positioning of the interbody graft. However, since the most recent radiographs with a mean follow-up time of 13.3 months were used for comparison in this study, subsidence should also be included as a potential limiting factor. 

The mean disc heights were significantly increased as a whole and at each segment as well. Dessicated, collapsed disc spaces are common in degenerative spine disease, and the addition of an 8 or 10 mm lordotic cage would certainly be expected to have this effect. This represents some key advantages of the MIS LIF technique in that the neural foramen is also enlarged through an indirect manner [38] with a large implant spanning the entire width of the endplate, leading to potentially less risk of subsidence [39] and subsequent maintained disc height.

As the role of MIS LIF in spinal deformity correction is further clarified through further research, it is important to keep in mind that the ultimate end goal should still be to reestablish spinopelvic harmony or the proportional relationships of one regional parameter to another as it relates to global spinopelvic alignment as spinopelvic harmony has been directly linked to a satisfactory postsurgical outcome as assessed by health-related quality of life instruments [11, 40]. Three basic radiographic targets to aim for in order to achieve spinopelvic harmony include: (1) SVA of <50 mm or T1-SI <0°, (2) pelvic tilt of <20°, and (3) LL = pelvic incidence ± 9° [11]. Attention to these three goals serves as the foundation for individual, patient-specific spinopelvic realignment in the sagittal plane, and even partial improvements of these parameters may translate to better clinical outcomes.

A limitation of this study is that an assessment of global sagittal and coronal balance was not possible in this patient population. Standing scoliosis radiographs are not routinely performed on patients without multisegmental degenerative spine disease and significant preoperative lumbar hypolordosis due to the risks from radiation exposure. Also, only 10° lordotic cages were used, and the use of a more lordotic angle and/or elective section of the ALL would potentially provide a greater degree of lordosis. 

## 5. Conclusion

Lumbar lordosis is an important component of overall global sagittal spinopelvic alignment, and MIS LIF via a retroperitoneal transpsoas approach may play an important role in modifying LL to better attain spinopelvic harmony. Segmental lordosis is significantly increased. Even though regional lordosis is not significantly increased, it is at least maintained. Disc heights were significantly increased, which led to indirect decompression of the neural foramina. Due to the nature of the operation, however, the indirect decompression may need to be accompanied by an additional section of the ALL and/or the addition of a more lordotic interbody cage to obtain a more robust increase in regional lumbar lordosis.

## Figures and Tables

**Table 1:** Demographics.

Parameter	Value
Total number of patients	35
Mean age at time of surgery (years)	61.3 (33–79)
Male : female ratio	11 : 24
Mean follow-up period (months)	13.3
Total number of levels fused	50
Levels fused per patient	1.42
